# A Systematic Review and Meta-Analysis on the Diagnostic Test Accuracy of Hepatorenal Index in Pediatric Metabolic Dysfunction-Associated Steatotic Liver Disease

**DOI:** 10.3390/diagnostics16050729

**Published:** 2026-03-01

**Authors:** Ratna Sutanto, Aristya Dewi Pratiwi, Callistus Bruce Henfry Sulay, Gilbert Sterling Octavius

**Affiliations:** 1 Pediatric Imaging Division, Department of Radiology, Faculty of Medicine, Universitas Pelita Harapan, Tangerang 15811, Indonesia; 2 Radiology Department, Faculty of Medicine, Universitas Pelita Harapan, Tangerang 15811, Indonesia

**Keywords:** hepatorenal index, metabolic dysfunction-associated steatotic liver disease, fatty liver, diagnostic accuracy, ultrasound

## Abstract

**Background**: Metabolic dysfunction-associated steatotic liver disease (MASLD), previously known as non-alcoholic fatty liver disease (NAFLD), is increasingly prevalent in children. However, reliable noninvasive diagnostic tools remain limited. The hepatorenal index (HRI) has been proposed as a quantitative ultrasound method to assess hepatic steatosis. This study aims to evaluate the diagnostic accuracy of HRI in detecting pediatric MASLD. **Methods**: A systematic review and meta-analysis were conducted on 13 September 2025, following PRISMA-DTA guidelines, with the protocol registered in PROSPERO (CRD420251146939). MEDLINE, PubMed, Cochrane Library, ScienceDirect, and Google Scholar were searched. Studies that assessed HRI against reference standards (MRI-PDFF or liver biopsy) in pediatric MASLD were included. Pooled diagnostic parameters were estimated using a bivariate random-effects model, with heterogeneity evaluated by I^2^ statistics and publication bias by funnel plot asymmetry. **Results**: Four studies involving 194 pediatric patients (47.9% MASLD), mostly male (57.7%), met the inclusion criteria. The suggested HRI cut-off varies from ≥1.215 to 1.99. The pooled sensitivity and specificity were 90% (95% CI 70–97) and 84% (95% CI 73–92), respectively, with an AUC of 0.91 (95% CI 0.88–0.93). Positive and negative likelihood ratios were 6 and 0.12, corresponding to post-test probabilities of 32% and 1%, respectively. No significant publication bias or heterogeneity was detected. **Conclusions**: Although HRI demonstrates strong diagnostic performance, it currently lacks sufficient discriminatory power to definitively confirm or exclude MASLD in pediatric populations and should therefore be regarded as a supportive rather than definitive diagnostic tool pending further high-quality validation studies.

## 1. Introduction

Previously considered a condition limited to adults, non-alcoholic fatty liver disease (NAFLD) was once thought to be rare in children. However, growing evidence indicates that pediatric NAFLD is increasingly prevalent, with a meta-analysis reporting a pooled mean prevalence of 7.6% in the general population and up to 34.2% among children attending obesity clinics [1]. In June 2023, a multisociety Delphi consensus agreed that NAFLD would be renamed as metabolic dysfunction-associated steatotic liver disease (MASLD) [2]. Shortly after, in January 2024, another multisociety Delphi consensus agreed on the term MASLD, while also acknowledging the term pediatric steatotic liver disease (SLD) [3]. From this point onward in this manuscript, NAFLD will be referred to as MASLD.

Since pediatric MASLD is usually a silent disease, the American Academy of Pediatrics (AAP) recommends screening for MASLD by measuring alanine transaminase (ALT) and aspartate aminotransferase (AST) levels in overweight children (body mass index [BMI] between the 85th and 94th percentile for age and sex) who present with additional risk factors, and in obese children (BMI ≥ 95th percentile) even when no risk factors are identified [4]. Liver ultrasound is the most widely used imaging method to screen for MASLD [5]. However, one pediatric study shows that both ALT and ultrasound (using ultrasound steatosis score) only have mediocre accuracy, and neither one of them is superior to the other. Combining both modalities does not improve the accuracy of screening, and a negative screening result does not exclude MASLD [6]. Since then, several quantitative ultrasound methods have been developed to detect MASLD, including ultrasound scores [7,8], shear-wave elastography, shear-wave dispersion, attenuation coefficient, backscatter coefficient, speed of sound, and H-scan, to name a few [9]. One systematic review in 2014 states that there is not enough evidence to support ultrasound to screen, diagnose, exclude, grade, or monitor fatty liver [10].

The hepatorenal index (HRI) is calculated manually as the ratio between the mean echogenicity of the liver and that of the right renal cortex, providing a quantitative sonographic measure of hepatic steatosis. This measurement was created to counter the subjectivity and low inter-observer variability of B-mode ultrasound in detecting hepatic steatosis [11]. Although a meta-analysis evaluating the diagnostic accuracy of HRI for detecting MASLD has been conducted in adults, no such analysis exists for the pediatric population. Therefore, this systematic review and meta-analysis aim to assess the diagnostic performance of HRI in identifying MASLD among children.

## 2. Materials and Methods

### 2.1. Eligibility Criteria

The authors conducted this study in accordance with the Preferred Reporting Items for Systematic Reviews and Meta-Analyses of Diagnostic Test Accuracy (PRISMA-DTA) guidelines [12]. A completed PRISMA 2020 checklist is provided as Supplementary Materials. The study protocol is registered in the International Prospective Register of Systematic Reviews (PROSPERO) under the identifier (CRD420251146939).

The studied population was all pediatric patients (≤18 years old) with MASLD. The diagnosis of MASLD requires evidence of hepatic steatosis and the exclusion of all other potential causes of liver fat accumulation, such as genetic or metabolic disorders (Wilson disease, uncontrolled diabetes or citrin deficiency), medications (amiodarone, valproic acids or corticosteroids), dietary causes, and infectious etiologies (hepatitis C genotype 3) [13]. The primary outcome of this study was to evaluate the sensitivity, specificity, positive likelihood ratio (LR+), negative likelihood ratio (LR−), positive predictive value (PPV), negative predictive value (NPV), and post-test probability of the HRI for detecting MASLD. The gold standard for diagnosing MASLD in pediatrics was liver biopsy [14]. However, due to the invasive nature of liver biopsies in a pediatric setting, magnetic resonance imaging with proton density fat fraction (MRI-PDFF) is an accepted alternative [15]. We excluded studies that used a different reference standard, included secondary MASLD caused by underlying diseases, or were only protocol reports. Consequently, our research question was formulated as follows: “In pediatric patients with confirmed MASLD, as determined by liver biopsy or MRI-PDFF, how accurately does the hepatorenal index (HRI) rule in or rule out MASLD?”.

The inclusion criteria were articles of any type, including cross-sectional, case–control, cohort, or randomized controlled trials, published in any language. The search was also conducted for grey literature, including theses, dissertations, and conference abstracts. The exclusion criteria included reviews, case series, case reports, or animal research. To ensure that the literature is saturated, review study citations are looked up. We also manually searched the cited literature to ensure that all pertinent studies were covered.

### 2.2. Search Strategy and Study Selection

The literature search started and ended on 13 September 2025. We searched five academic databases: MEDLINE, Cochrane Library, PubMed, Science Direct, and Google Scholar. The keywords used were related to the diagnostic tool (“hepatorenal index”), the conditions under study (“fatty liver”, “hepatic steatosis”, “metabolic dysfunction-associated steatotic liver disease”, and “non-alcoholic fatty liver disease”), and the population (“children”, “adolescent”, and “pediatric”). Supplementary Table S1 lists the Medical Subject Headings (MeSH) terms for each database. We purposely avoided using any keywords that mentioned “sensitivity,” “specificity,” or other terms related to diagnosis, because doing so could cause relevant research to be overlooked [16]. All records were entered into the Rayyan program, which performed manual screening and automatic duplicate detection [17]. Three authors (ADP, CBHS, and GSO) conducted the initial search and imported all data into Rayyan, while a separate author (RS) cross-checked the searches. Each paper underwent independent evaluation by the first three authors, and any disagreements were resolved through group discussion and expert judgment by (RS). When studies used overlapping datasets, the data providing the most comprehensive information were selected.

### 2.3. Data Extraction and Quality Assessment

Three authors (ADP, CBHS, and GSO) independently extracted the data, while RS verified its accuracy. We gathered pertinent data, including study identity (author and publication year), study characteristics (location, study design, age of the participants, and study term), and information regarding ultrasonography (machines used, techniques, the probes used, and region of interest [ROI] placements for the HRI).

The risk of bias was assessed using the Revised Tool for the Quality Assessment of Diagnostic Accuracy Studies (QUADAS-2). This tool does not provide official cut-off scores; instead, the risk of bias is presented graphically [18]. Three reviewers evaluated the scale independently (RS), and any disagreements were resolved internally and through an expert decision (RS) until a consensus was reached. We contacted the associated authors by email to see if there was any missing or incomplete data.

### 2.4. Data Synthesis

For each study, sensitivity, specificity, LR+, LR−, PPV, and NPV were calculated when these values were not explicitly reported in the original article [19]. Before conducting the meta-analysis, model diagnostics were assessed through graphical evaluation of residual-based goodness of fit, tests of bivariate normality, and analyses for influence and outlier detection. Sensitivity analyses were performed to confirm the impact of any potential outliers. The degree of interdependence between studies was illustrated using a bivariate box plot. A bivariate model of sensitivity and specificity was then applied to estimate both individual study values and pooled sensitivity and specificity [20]. Using the hierarchical summary receiver operating characteristic (HSROC), a summary receiver operating characteristic (SROC) would be utilized to show and highlight the trade-off between sensitivity and specificity [21]. The area under the curve (AUC) was calculated to assess diagnostic performance, with values of 0.9–1.0 reflecting excellent accuracy, 0.8–0.9 very good accuracy, 0.7–0.8 good accuracy, 0.6–0.7 fair accuracy, and 0.5–0.6 indicating poor diagnostic accuracy [22]. Heterogeneity was assessed using the I^2^ statistic, where a value of 0% indicates no observable heterogeneity, and values exceeding 50% are generally interpreted as representing substantial heterogeneity [23]. To evaluate potential publication bias, we performed a linear regression test for funnel plot asymmetry, where a slope coefficient below 0.1 was considered indicative of significant asymmetry. Post-test probability was then estimated using a Fagan nomogram derived from the likelihood ratio scattergram and Bayes’ theorem. Because no direct data were available on the pre-test probability of MASLD in otherwise healthy children, we adopted a value of 7.6% based on a meta-analysis of MASLD prevalence in healthy pediatric populations [1]. It should be noted that the post-test probabilities were calculated using a pre-test probability of 7.6%, reflecting MASLD prevalence in the general pediatric population [1]. In higher-risk settings, such as pediatric obesity clinics where prevalence may approach 34.2% [1], the same likelihood ratios would yield substantially different post-test probabilities. Therefore, clinicians should interpret HRI results within the context of the baseline risk of their specific patient population. An arbitrary threshold was defined as an LR− value below −0.1 and an LR+ value above +10, representing a significant change in the probability ratio [24]. Additionally, probability-modifying plots and predictive values were generated, where curves approaching the (0, 1) point indicate test outcomes with stronger positive predictive value, while curves trending toward the (1, 0) point reflect test outcomes with greater negative predictive value [25]. The meta-analysis was performed using STATA software (Version 17.0, StataCorp, College Station, TX, USA) with the “midas” command [26].

## 3. Results

**Figure 1:**
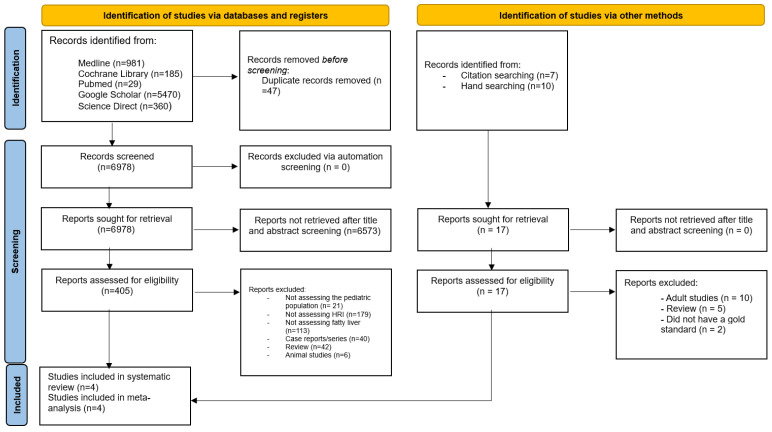
PRISMA flowchart for selection of included studies.

A total of 7025 articles were initially identified, with 47 duplicates removed, leaving 6978 unique records for screening. After reviewing titles and abstracts, 6573 articles were excluded, and ultimately, four studies met the inclusion criteria and were incorporated into the meta-analysis [27,28,29,30] (Figure 1).

Notable exclusions include studies that utilize HRI as a diagnostic method (i.e., have no reference standard) [31,32,33,34,35,36], two studies that enroll patients with underlying chronic liver diseases, which do not qualify for the definition of MASLD [37,38], and one study that uses MRI fat fraction only (i.e., without PDFF sequence) [39]. Lastly, one study does not assess the diagnostic test accuracy between HRI and MRI-PDFF due to the non-significance in correlation. After two unreplied emails, this study is regrettably excluded [40].

**Figure 2:**
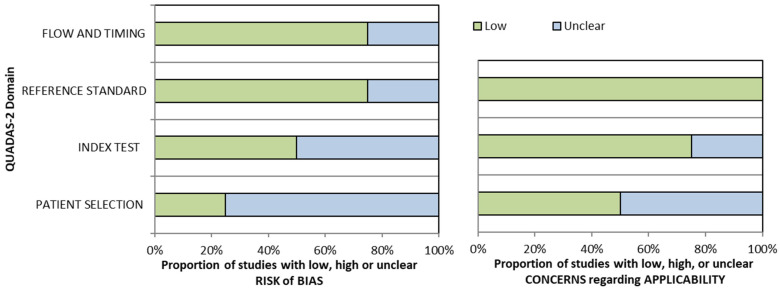
QUADAS-2 graphical representation of the risk-of-bias.

All studies are retrospective in nature [27,28,29], except for one prospective study [30]. Only one study explicitly mentioned that the sampling was done consecutively [30], while the rest did not state the sampling methodology. Across all four studies, there were 93 children with MASLD out of 194 patients, ranging from 0.3 to 18 years old, with mostly male (57.7%). Three studies were done in the United States of America (USA) [27,28,30], with only one study done in Italy [29]. Amongst the three studies that report the body mass index (BMI), most of the patients are in the overweight-obese criteria [27,28,30]. All of the studies use MRI-PDFF as the gold standard, with a varying cut-off from ≥5% to 6%. The time that elapses between ultrasound and MRI-PDFF ranges from being done on the same day [30] to a median of 38 days [28] (Table 1). Two studies placed the region of interest (ROI) at the same depth for both the liver and the kidney [28,30], whereas one study applied a freehand ROI without clearly specifying that the measurements were taken at an equivalent depth [27]. The other study uses an automated software (EzHRITM), which automatically recommends the ROI placement [29]. The suggested cut-off varies from ≥1.215 [29] to 1.99 [28] (Table 2). All studies suffer from some risks of bias as they face unclear risks in almost all aspects (Table 3 and Figure 2).

The model diagnostics are shown in Supplementary Figure S1, where no studies appear to be outliers with a reasonable goodness-of-fit. However, the bivariate normality assumption is not fulfilled, and two studies [27,29] seem to be more influential than others with a Cook’s distance of >0.5. The bivariate box plot showed a skewness of the test performance measures toward a higher sensitivity with lower specificity, providing indirect evidence of some threshold variability (Supplementary Figure S2).

**Figure 3:**
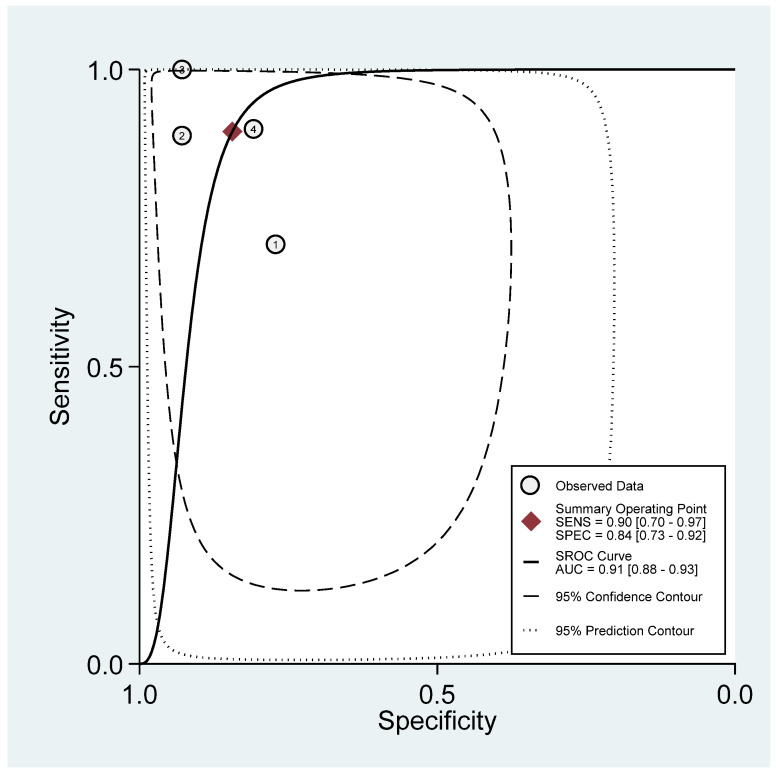
Summary receiver operating curve with confidence and prediction regions.

**Figure 4:**
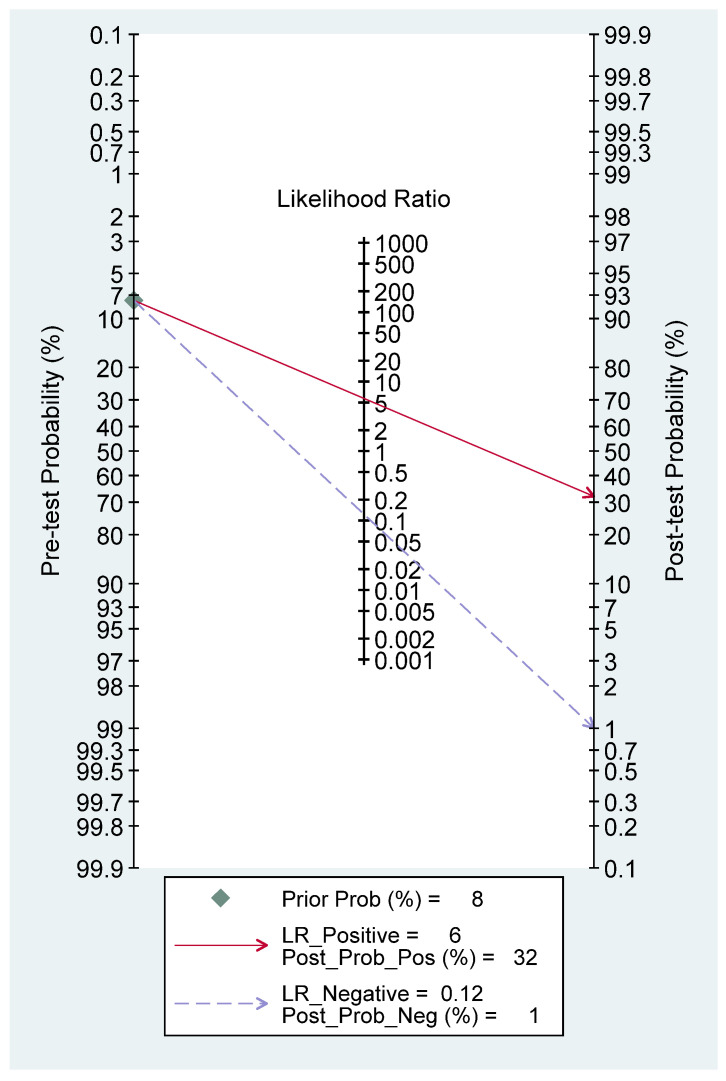
Fagan’s nomogram of HRI in detecting pediatric MASLD.

**Figure 5:**
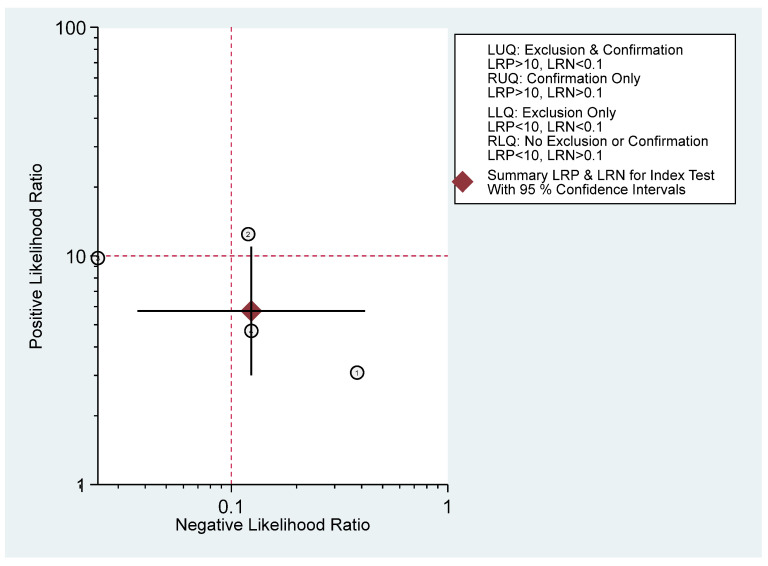
Likelihood ratio scattergram.

**Figure 6:**
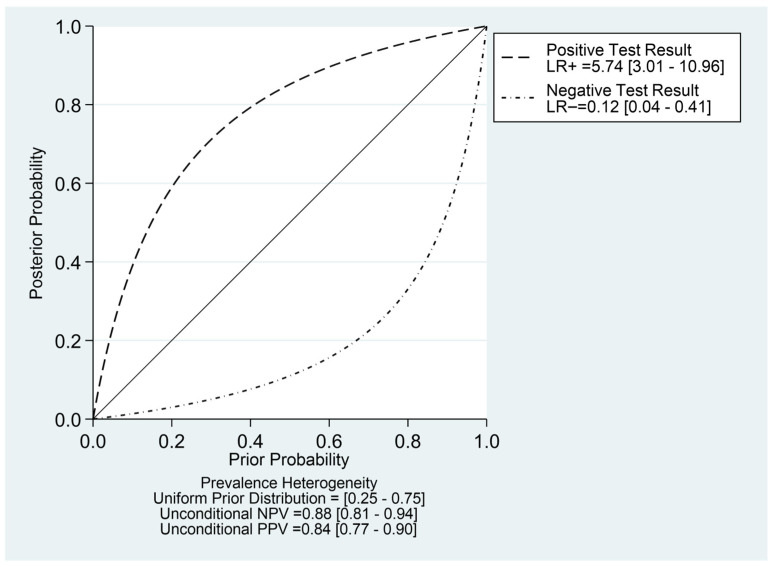
Probability Modifying Plot.

Figure 3 shows that the combined sensitivity is 90% (95% confidence interval [CI] 70–97) and specificity of 84% (95% CI 73–92). The combined SROC yields an area under the curve of 0.91 (95% CI 0.88–0.93), indicating that the HRI has very good diagnostic accuracy in diagnosing pediatric MASLD. Supplementary Figure S3 presents the paired forest plot, which demonstrates that the observed sensitivities and specificities of the four included studies vary in both magnitude and precision. In contrast, the empirical Bayes (EB) estimates are consistently drawn toward the pooled mean, reflecting the shrinkage effect of the bivariate model. Studies with wider confidence intervals, particularly for sensitivity, show greater adjustment toward the overall mean, indicating lower precision or smaller sample sizes. The I^2^ value is 0% (95% CI 0–100) with a *p*-value of 0.271, indicating non-significant undetected heterogeneity. Figure 4 displayed Fagan’s Nomogram, showing that the LR+ is 6 with a 32% post-test probability (a roughly 24% increase from the baseline) while the LR− is 0.12 with a 1% post-test probability (a roughly 7% decrease from the baseline). The likelihood ratio scattergram placed the summary point of likelihood ratios in the right lower quadrant, meaning that HRI cannot be used to exclude or confirm pediatric MASLD (Figure 5). The probability modifying plot tends to slightly favour the (1, 0) line, indicating a more informative negative result. The combined negative predictive value is 0.88 (95% CI 0.81–0.94), and the positive predictive value is 0.84 (95% CI 0.77–0.90) (Figure 6).

The linear regression test for funnel plot asymmetry yields a *p*-value of 0.19, indicating insignificant asymmetry, and thus there is a low chance of publication bias (Supplementary Figure S4).

## 4. Discussion

This systematic review and meta-analysis includes four studies enrolling 194 patients, with 47.9% children suffering from MASLD. The hepatorenal index demonstrated high diagnostic performance, with a sensitivity of 91% and specificity of 84%, corresponding to an LR+ of 6 and an LR− of 0.12. However, the likelihood ratio scattergram indicated that the HRI alone is insufficient to either confirm or exclude pediatric MASLD. A meta-analysis of HRI in adults included 13 studies with 1496 cases, which reported a pooled sensitivity of 87% and a specificity of 89%, with an AUC of 0.94, an LR+ of 7.6, and an LR− of 0.15. Their subgroup analysis also shows that the diagnostic performance of HRI is influenced by publication year, ROI shape, sample size, and HRI thresholds [41]. While our results are comparable, the meta-analysis done in the adult cohort enrolled more studies and patients, which may affect our results.

Metabolic dysfunction-associated steatotic liver disease encompasses a spectrum of liver abnormalities, ranging from uncomplicated hepatic steatosis to non-alcoholic steatohepatitis (NASH). The pathogenesis of MASLD is not yet completely elucidated, pointing to a multifactorial component with a complex network of interactions involving environmental, genetic, and epigenetic components [5]. The newest consensus states that pediatric MASLD needs to meet cardiometabolic criteria, as well as the exclusion of other diseases [3], which leads to the exclusions of two studies that included children with Wilson disease, hepatitis C, primary sclerosing cholangitis, cirrhosis, autoimmune hepatitis, α1-antitrypsin deficiency, and total parenteral nutrition-related liver disease, to name a few [37,38]. Therefore, under the new consensus [3], these studies enroll patients with MASLD overlap, cryptogenic SLD, or other single etiology of pediatric SLD, which may contribute to the heterogeneity of our meta-analysis.

It is important to recognize the practical advantages of HRI. The technique is noninvasive, radiation-free, relatively inexpensive, and widely accessible compared with MRI-PDFF or liver biopsy. Unlike qualitative B-mode assessment, HRI provides a semi-quantitative measurement that reduces subjectivity by using the renal cortex as an internal reference [11]. In this meta-analysis, HRI demonstrated a pooled AUC of 0.91, reflecting very good diagnostic accuracy. These characteristics make HRI an attractive adjunctive screening tool in resource-limited settings or in institutions where advanced imaging modalities are not readily available.

There are several caveats in using HRI in daily clinical practice. Firstly, HRI cannot be used in patients with right renal agenesis, right ectopic kidney, right nephrectomy, renal atrophy, or chronic kidney disease, since the measurement cannot be done at all, or the accuracy will be limited [42,43]. Secondly, the overall gain setting and operating frequencies will affect the accuracy of HRI, as proper visualization of liver parenchyma is needed to visualize the areas indicated for ROI placement [43,44]. Thirdly, the accuracy of HRI measurement is highly dependent on the quality of the acquired B-mode image, which is an issue in retrospective studies that may not obtain their images specifically for this study [43]. The fourth limitation is the varying measurement from the skin surface to the liver capsule distance, which contributes to high failure rates of HRI, especially in obese patients [42,43]. The variability in HRI thresholds has important implications for clinical implementation. Differences in ultrasound equipment, gain settings, ROI size and placement, BMI distribution, and study design may contribute to threshold heterogeneity [41,43,44]. The skewness observed in the bivariate box plot suggests a possible threshold effect, where higher sensitivity may be achieved at the expense of specificity. Without a standardized cut-off value validated across diverse pediatric populations, the generalizability of a single diagnostic threshold remains limited. Therefore, harmonization of acquisition protocols and external validation studies is essential before routine clinical adoption. From a practical standpoint, inter-operator variability is an important consideration. Among the included studies, Hajibonabi et al. [28] reported good interobserver agreement for HRI measurement, suggesting acceptable reproducibility in experienced hands. The HRI was originally developed to reduce the subjectivity inherent in qualitative B-mode assessment [11]. Nevertheless, ROI placement technique and image acquisition parameters may still influence reproducibility [43,45]. Standardized acquisition protocols and operator training may therefore be necessary to ensure consistent performance across institutions. The last limitation is that renal cortical backscatter anisotropy produces inaccurate liver fat estimates [46]. Another pediatric-specific consideration is the potential influence of age-related changes in renal cortical echogenicity. Previous sonographic studies have demonstrated that renal parenchymal echogenicity varies with age, particularly in infants and young children, gradually approaching adult patterns over time [47]. Because HRI relies on the renal cortex as an internal echogenic reference, age-dependent differences in renal echotexture may theoretically influence liver-to-kidney contrast ratios and thus HRI values. None of the included studies performed age-stratified analyses to evaluate this potential effect. Future pediatric studies should consider age-adjusted reference values or subgroup analyses to determine whether renal maturation influences HRI performance.

This meta-analysis has several limitations in addition to the caveats previously mentioned. The first limitation is that not all included studies performed subgroup analyses comparing obese and non-obese children. One pediatric study finds that HRI is higher in obese pediatric patients with obstructive sleep apnea compared to non-obese pediatric patients [35]. Another study in adult patients demonstrated that the diagnostic performance of HRI varies among individuals with BMI values ≥30 and ≥35, suggesting the presence of a BMI-dependent threshold effect [48]. The second limitation is that HRI can only detect MASLD, but this meta-analysis cannot detect its use in NASH, hepatic fibrosis, or cirrhosis. The third limitation is the different cut-off used for MRI-PDFF across studies. There is still no consensus on the optimal cut-off to detect hepatic steatosis, with studies citing values from 5% [45] to 6% [15]. This variability in MRI-PDFF thresholds (ranging from ≥5% to ≥6%) underscores the need for standardization of the reference standard when evaluating ultrasound-based fat quantification tools. Şendur and Şendur (2022) emphasized that inconsistent MRI-PDFF cut-offs may lead to artificial inflation or attenuation of diagnostic performance metrics, thereby complicating comparisons across studies [49]. Since MRI-PDFF serves as the reference standard in all included studies, even small variations in threshold definitions may influence sensitivity and specificity estimates. Although MRI-PDFF is widely accepted as a noninvasive surrogate for histologic steatosis [15,45], harmonization of acquisition protocols and consensus cut-off values is essential to ensure accurate benchmarking of emerging ultrasound techniques such as HRI. The fourth limitation concerns the method of ROI acquisition. ROIs can be manually drawn using software such as ImageJ or directly from the Picture Archiving and Communication System (PACS). One study reported no significant difference between these two manual approaches [50]. However, evidence comparing automated ROI measurement tools, such as EzHRI™, with manually derived HRI values remains limited and inconclusive. Additionally, vendor variability represents another potential source of measurement heterogeneity. The included studies utilized ultrasound systems from multiple manufacturers, including Canon, Philips, GE, and Samsung. Differences in beamforming algorithms, transducer characteristics, grayscale mapping, and proprietary image post-processing may influence echogenicity measurements and, consequently, HRI values. Without cross-vendor calibration or standardized phantom validation, it remains uncertain whether identical HRI thresholds can be universally applied across platforms. Future multicenter studies should address inter-vendor reproducibility to enhance generalizability. The fifth limitation is that none of the included studies used liver biopsy as the reference standard. This issue reflects the reluctance to employ an invasive procedure in a largely asymptomatic pediatric population. Nevertheless, current guidelines recommend performing a liver biopsy only in overweight or obese children under eight years old with suspected MASLD, when there is a strong suspicion of advanced disease, or when alternative diagnoses need to be ruled out [51]. Finally, the most evident limitation is the small number of included studies (*n* = 4) and the relatively modest cumulative sample size (194 children). This limited evidence base reduces the precision of pooled estimates and restricts subgroup analyses, including BMI-stratified or age-stratified performance assessments. Although small pediatric sample sizes are common in the literature [13,52], additional large-scale, multicenter prospective studies are required before HRI can be confidently integrated into standardized pediatric diagnostic pathways.

Despite the limitations, there are some notable strengths to this study. To our knowledge, this is the first meta-analysis of HRI in pediatric MASLD patients. This meta-analysis also points out that there is no current evidence of HRI in diagnosing or excluding MASLD in a pediatric patient, despite some other studies already employing HRI as a diagnostic tool [31,32,33,34,35,36]. Although MRI-PDFF has limitations such as long acquisition time and limited availability in some centers, and liver biopsy is invasive and subject to sampling error [53], these drawbacks do not justify the routine use of HRI for diagnosing pediatric MASLD, given the current lack of sufficient supporting evidence. Finally, this meta-analysis highlights the existing limitations and weaknesses of HRI in diagnosing pediatric MASLD, providing valuable insights that future studies can build upon to design more robust and methodologically sound research.

## 5. Conclusions

The hepatorenal index demonstrates promising diagnostic performance for detecting MASLD in children, showing high pooled sensitivity and specificity. However, the likelihood ratio analysis reveals that HRI alone cannot conclusively confirm or exclude pediatric MASLD. The limited number of available studies, small sample sizes, heterogeneity in reference standards, and methodological variability restrict the generalizability of these findings. Despite these limitations, this meta-analysis provides an essential foundation for future research exploring noninvasive diagnostic tools for pediatric MASLD. Further large-scale, multicenter studies with standardized protocols, BMI-stratified analyses, and comparison against histopathologic or advanced imaging standards are warranted. Until stronger evidence emerges, HRI should be used cautiously as an adjunct, not a standalone, diagnostic modality.

## Figures and Tables

**Table 1:** Descriptive findings of included studies.

Author (Year)	Study Design	Sampling	Country	Age (Years)	Female Sex (Total)	Patients with Steatosis (Total)	Body Mass Index	Personnel Performing the Ultrasound	Analysis of HRI	Image Reviewer	Gold Standard	Definition of Hepatosteatosis on the Gold Standard	Time Elapsed Between Ultrasound and Gold Standard	Ultrasound Machine
Frankland (2022) [27]	Retrospective	Not mentioned	USA	Mean = 11.6 ± 4.7; Range = 0.3–18	27 (69)	34 (69)	Mean percentile = 76.5 ± 29.8%; Range = 1–99	Ultrasound technologists	PACS	One pediatric radiologist	MRI-PDFF within 3 months of each other	MRI PDFF values ≥ 6%.	9.5 ± 9.2 days (0–88 days)	Aplio 500 or Aplio i800 (Canon Medical Systems, Tustin, CA, USA) US systems using a curved 6C1 transducer
Hajibonabi (2024) [28]	Retrospective	Not mentioned	USA	Median = 13 (10–15)	16 (41)	27 (41)	Not mentioned	Pediatric ultrasound technologists	PACS	Three radiologists	MRI-PDFF within 3 months of each other	MRI PDFF values ≥ 5.2%.	38 (7–52 days)	Philips ultrasound machine (EPIQ and IU22, Philips Healthcare, Bothell, WA, USA) using curved transducers (C5-1 or C8-5) or on a GE ultrasound machine (LogiQ E10, GE Healthcare, Waukesha, WI, USA) using curved and linear transducers (C1-6, C2-9, or L2-9)
Polti (2023) [29]	Retrospective	Not mentioned	Italy	13 (11–14) in non-fatty cohort, 11 (10–13) in fatty cohort	17 (36)	22 (36)	27.9 (26.2–28.8) in non-fatty liver patients, 26.2 (23.3–31.4) in fatty liver patients	Two radiologists	EzHRI^TM^	None	MRI-PDFF	MRI PDFF values ≥ 5.6%.	Not mentioned	RS85 A, Samsung Medison, Seoul, Republic of Korea, with a curved array transducer
D’Hondt (2021) [30]	Prospective	Consecutive	USA	13 ± 3 (range, 7–17)	22 (48)	10 (48)	22.25 ± 6 (range, 14.5–48.1)	One pediatric radiologist	PACS	None	MRI-PDFF on the same day or one day before or after the ultrasound	MRI PDFF ≥ 5%.	On the same day, or one day before or after the ultrasound	EPIQ-7G, Philips Healthcare) equipped with a curved-array transducer in the frequency range of 1–5 MHz (C5–1, Philips Healthcare)

USA, United States of America; PACS, Picture Archiving and Communication System; MRI-PDFF, Magnetic Resonance Imaging-Proton Density Fat Fraction.

**Table 2:** Diagnostic test accuracy of each study.

Author (Year)	ROI Placement	HRI Cut-Off	TP	FP	TN	FN
Frankland (2022) [27]	Small circular and large freehand ROIs in the right renal cortex and adjacent liver on longitudinal and transverse images	>1.75	24	8	27	10
Hajibonabi (2024) [28]	20–30 mm^2^ ROIs in renal cortex and liver segment VI at the same depth, avoiding medulla, vessels, ducts, and artifacts	1.99	24	1	13	3
Polti (2023) [29]	ROI placement suggested automatically by EzHRI	≥1.215	22	1	13	0
D’Hondt (2021) [30]	50 mm^2^ (25–80 mm^2^) ROIs at the same depth for liver and kidney, lateral proximity, in a homogeneous liver area, avoiding vessels, ducts, shadowing, or masses	1.48	9	9	38	1

ROI, Region of Interest; TP, True positive; FP, False positive; TN, True negative; FN, False negative.

**Table 3:** QUADAS-2 results of each study.

Study	Risk of Bias				Applicability Concerns			Conclusions
	Patient Selection	Index Test	Reference Standard	Flow and Timing	Patient Selection	Index Test	Reference Standard	
Frankland (2022) [27]	?	☺	☺	☺	☺	☺	☺	At risk of bias
Hajibonabi (2024) [28]	?	☺	☺	☺	?	?	☺	At risk of bias
Polti (2023) [29]	?	?	?	?	☺	☺	☺	At risk of bias
D’Hondt (2021) [30]	☺	?	☺	☺	?	☺	☺	At risk of bias

☺ Low Risk; ? Unclear Risk.

## Data Availability

All data are provided under Supplementary Materials.

## Supplementary Materials

The following supporting information can be downloaded at: https://www.mdpi.com/article/10.3390/diagnostics16050729/s1, Figure S1: Model diagnostics of each study. In this figure, study 1 belongs to Frankland (2022) [27], study 2 belongs to Hajibonabi (2024) [28], study 3 belongs Polti (2023) [29], and study 4 belongs to D’Hondt (2021) [30]; Figure S2: Bivariate boxplot of each study; Figure S3: Paired forest plot showing observed (open circles, dashed lines) and empirical Bayes–predicted (filled circles, solid lines) sensitivities (left) and specificities (right) for each included study. Vertical solid lines represent the pooled mean estimates. The empirical Bayes predictions demonstrate shrinkage toward the overall mean, reflecting stabilization of study-level estimates compared with the observed data; Figure S4: Linear regression test of funnel plot asymmetry; Table S1: Medical subject heading (MeSH) terms used in each database; Table S2: PRISMA 2020 Checklist [54].

## Abbreviations

The following abbreviations are used in this manuscript:

AAP	American Academy of Pediatrics
AUC	Area Under the Curve
ALT	Alanine Transaminase
AST	Aspartate Aminotransferase
BMI	Body Mass Index
CI	Confidence Interval
FN	False Negative
FP	False Positive
HRI	Hepatorenal Index
HSROC	Hierarchical Summary Receiver Operating Characteristic
I^2^	Inconsistency Index
LR+	Positive Likelihood Ratio
LR−	Negative Likelihood Ratio
MASLD	Metabolic Dysfunction-Associated Steatotic Liver Disease
MRI-PDFF	Magnetic Resonance Imaging–Proton Density Fat Fraction
NAFLD	Non-Alcoholic Fatty Liver Disease
NASH	Non-Alcoholic Steatohepatitis
NPV	Negative Predictive Value
PACS	Picture Archiving and Communication System
PDFF	Proton Density Fat Fraction
PPV	Positive Predictive Value
PRISMA-DTA	Preferred Reporting Items for Systematic Reviews and Meta-Analyses of Diagnostic Test Accuracy
PROSPERO	International Prospective Register of Systematic Reviews
QUADAS-2	Quality Assessment of Diagnostic Accuracy Studies, Version 2
ROI	Region of Interest
SLD	Steatotic Liver Disease
SROC	Summary Receiver Operating Characteristic
TP	True Positive
TN	True Negative
USA	United States of America
