# Supplementary material for: A Systematic Review and Meta-Analysis on the Diagnostic Test Accuracy of Hepatorenal Index in Pediatric Metabolic Dysfunction-Associated Steatotic Liver Disease
Source: Diagnostics (Basel). 2026 Mar 1;16(5):729. doi: 10.3390/diagnostics16050729 (PMC12985247; doi:10.3390/diagnostics16050729)
Supplement: Supplementary file 1 [file diagnostics-16-00729-s001.zip › Supplementary Figures and Tables HRI.pdf]

Supplementary Table S1. Medical subject heading (MeSH) terms used in each database

Database	Medical Subject Heading	Number of studies found
Pubmed	"Hepatorenal"[All Fields] AND ("abstracting and indexing"[MeSH Terms] OR ("abstracting"[All Fields] AND "indexing"[All Fields]) OR "abstracting and indexing"[All Fields] OR "index"[All Fields] OR "indexed"[All Fields] OR "indexes"[All Fields] OR "indexing"[All Fields] OR "indexation"[All Fields] OR "indexations"[All Fields] OR "indexe"[All Fields] OR "indexer"[All Fields] OR "indexers"[All Fields] OR "indexs"[All Fields]) AND ("fatty liver"[MeSH Terms] OR ("fatty"[All Fields] AND "liver"[All Fields]) OR "fatty liver"[All Fields] OR ("hepatic"[All Fields] AND "steatosis"[All Fields]) OR "hepatic steatosis"[All Fields] OR ("fatty liver"[MeSH Terms] OR ("fatty"[All Fields] AND "liver"[All Fields]) OR "fatty liver"[All Fields]) OR (("metabolic"[All Fields] OR "metabolical"[All Fields] OR "metabolically"[All Fields] OR "metabolics"[All Fields] OR "metabolism"[MeSH Terms] OR "metabolism"[All Fields] OR "metabolisms"[All Fields] OR "metabolism"[MeSH Subheading] OR "metabolities"[All Fields] OR "metabolization"[All Fields] OR "metabolize"[All Fields] OR "metabolized"[All Fields] OR "metabolizer"[All Fields] OR "metabolizers"[All Fields] OR "metabolizes"[All Fields] OR "metabolizing"[All Fields]) AND ("associate"[All Fields] OR "associated"[All Fields] OR "associates"[All Fields] OR "associating"[All Fields] OR "association"[MeSH Terms] OR "association"[All Fields] OR "associations"[All Fields]) AND ("non alcoholic fatty liver disease"[MeSH Terms] OR ("non alcoholic"[All Fields] AND "fatty"[All Fields] AND "liver"[All Fields] AND "disease"[All Fields]) OR "non alcoholic fatty liver disease"[All Fields] OR ("fatty"[All Fields] AND "liver"[All Fields] AND "disease"[All Fields]) OR "fatty liver disease"[All Fields])) OR ("non alcoholic fatty liver disease"[MeSH Terms] OR ("non alcoholic"[All Fields] AND "fatty"[All Fields] AND "liver"[All Fields] AND "disease"[All Fields]) OR "non alcoholic fatty liver disease"[All Fields] OR ("non"[All Fields] AND "alcoholic"[All Fields] AND "fatty"[All Fields] AND "liver"[All Fields] AND "disease"[All Fields]) OR "non alcoholic fatty liver disease"[All Fields])) AND ("paediatrics"[All Fields] OR "pediatrics"[MeSH Terms] OR "pediatrics"[All Fields] OR "paediatric"[All Fields] OR "pediatric"[All Fields] OR ("child"[MeSH Terms] OR "child"[All Fields] OR "children"[All Fields] OR "child s"[All Fields] OR "children s"[All Fields] OR "childrens"[All Fields] OR "childs"[All Fields]) OR ("adolescences"[All Fields] OR "adolescency"[All Fields] OR "adolescent"[MeSH Terms] OR "adolescent"[All Fields] OR "adolescence"[All Fields] OR "adolescents"[All Fields] OR "adolescent s"[All Fields]))	29
Medline	Hepatorenal index	981
Cochrane Library	(hepatorenal index):ti,ab,kw	185
Google Scholar	hepatorenal index children "fatty liver"	5470
Science Direct	(Hepatorenal index) AND (Children OR Pediatrics) AND (Fatty Liver OR Hepatic Steatosis) [Research articles only]	360

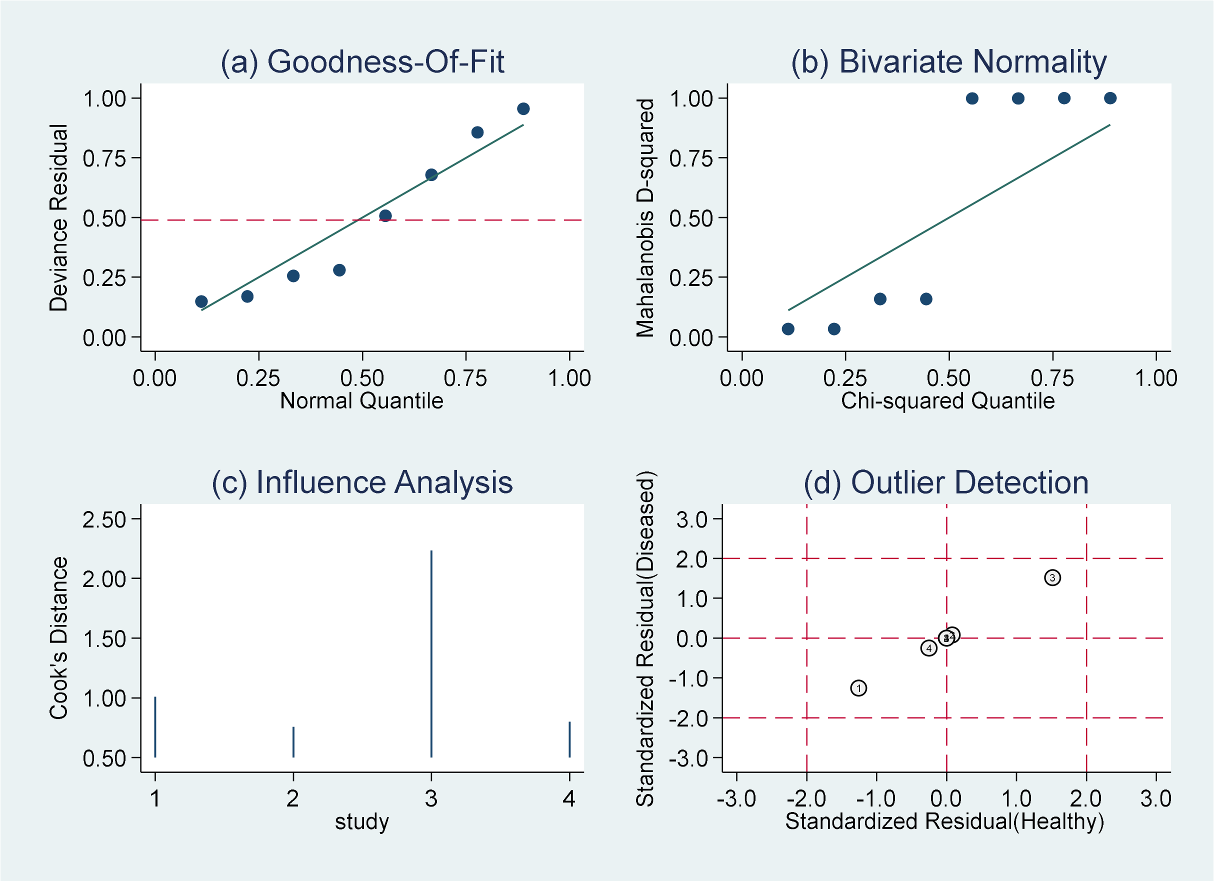

Supplementary Figure S1. Model diagnostics of each study. In this figure, study 1 belongs to Frankland (2022)[(1)], study 2 belongs to Hajibonabi (2024)[(2)], study 3 belongs Polti (2023)[(3)], and study 4 belongs to D'Hondt (2021)[(4)]

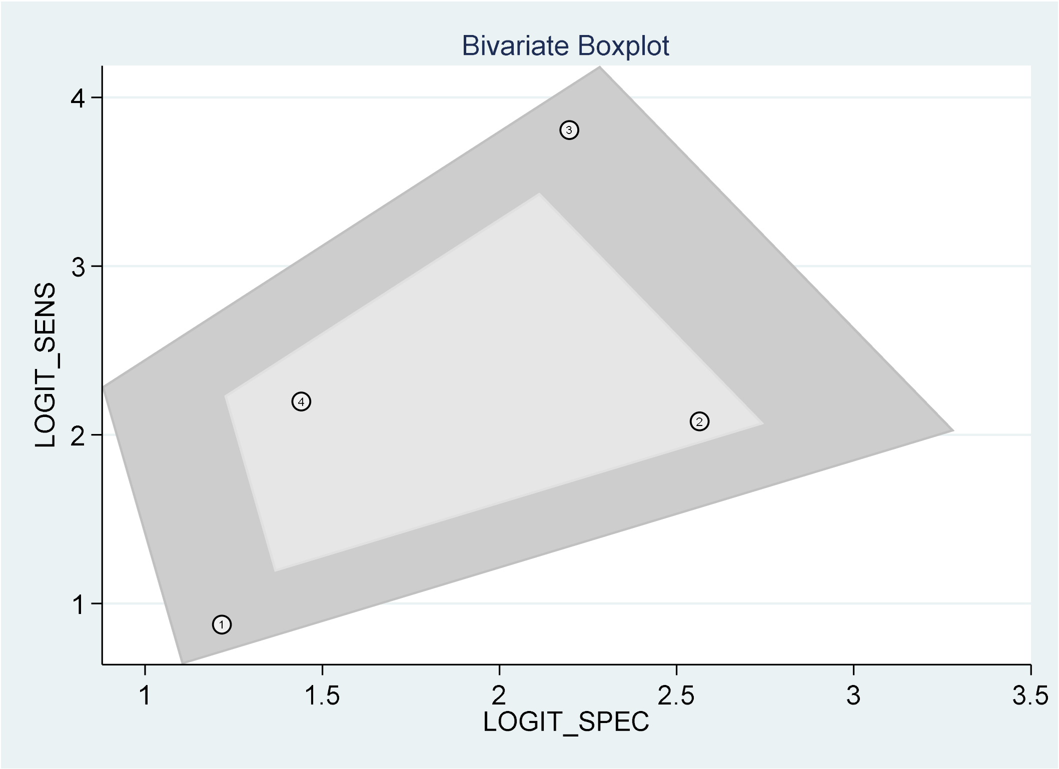

Supplementary Figure S2. Bivariate boxplot of each study

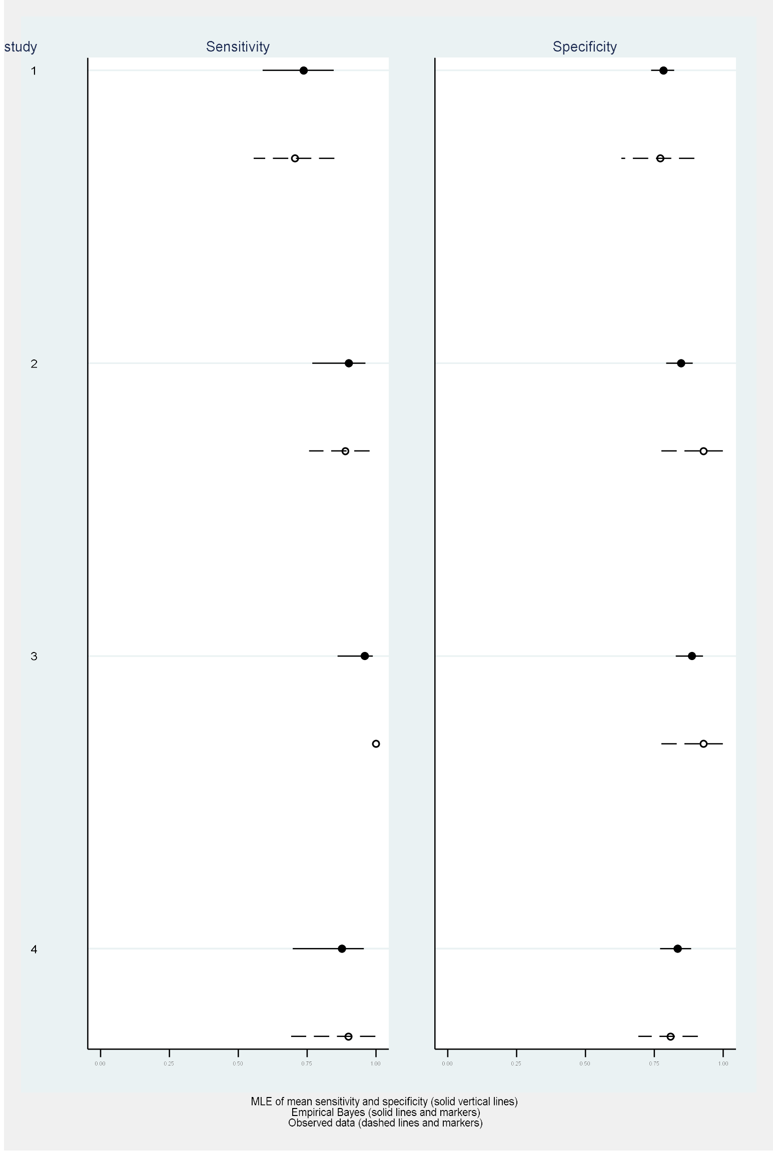

Supplementary Figure S3. Paired forest plot showing observed (open circles, dashed lines) and empirical Bayes–predicted (filled circles, solid lines) sensitivities (left) and specificities (right) for each included study. Vertical solid lines represent the pooled mean estimates. The empirical Bayes predictions demonstrate shrinkage toward the overall mean, reflecting stabilization of study-level estimates compared with the observed data.

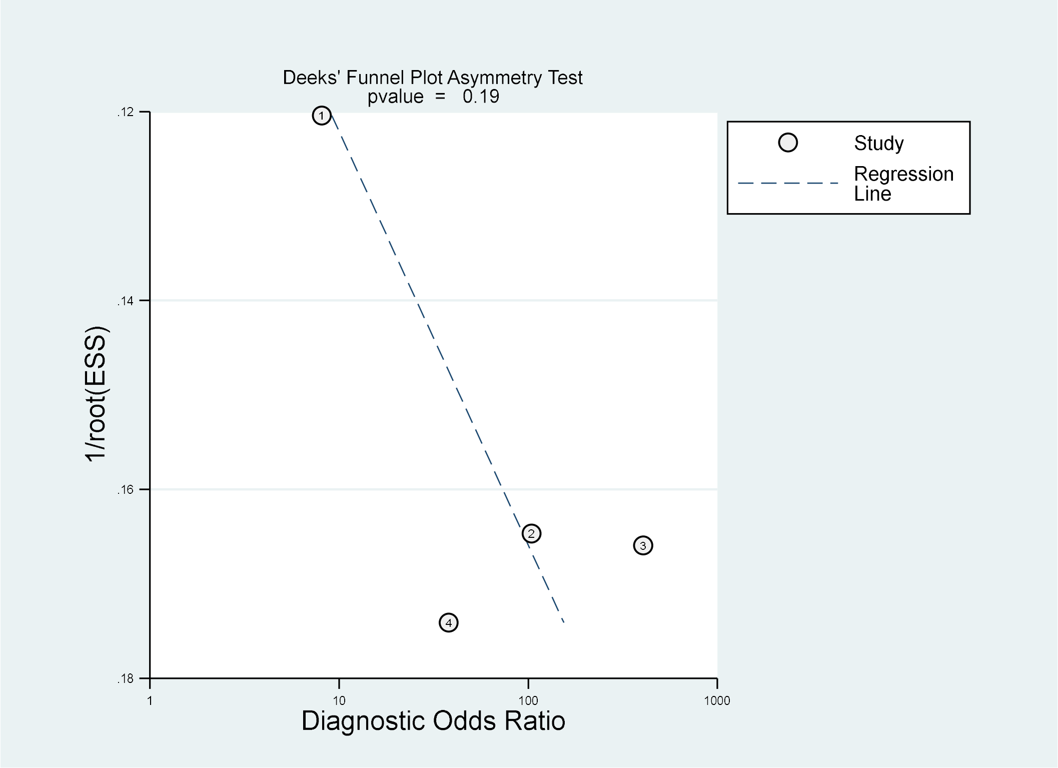

yb	Coefficient	Standard error	t	P>|t|	[95% confidence interval]
Bias	52.6	26.6	1.98	0.19	-61.9 – 167.0
Intercept	-4.1	4.0	-1.02	0.41	-21.4 – 13.2

Supplementary Figure S4. Linear regression test of funnel plot asymmetry

Supplementary References

1. Frankland MP, Dillman JR, Anton CG, Coley BD, Nasser MP, O'Hara SM, et al. Diagnostic performance of ultrasound hepatorenal index for the diagnosis of hepatic steatosis in children. Pediatr Radiol. 2022;52(7):1306-13.
2. Hajibonabi F, Riedesel EL, Taylor SD, Linam LE, Alazraki AL, Zhang C, et al. Ultrasound-estimated hepatorenal index: diagnostic performance and interobserver agreement for pediatric liver fat quantification. Pediatr Radiol. 2024;54(10):1653-60.
3. Polti G, Frigerio F, Del Gaudio G, Pacini P, Dolcetti V, Renda M, et al. Quantitative ultrasound fatty liver evaluation in a pediatric population: comparison with magnetic resonance imaging of liver proton density fat fraction. Pediatr Radiol. 2023;53(12):2458-65.
4. D'Hondt A, Rubesova E, Xie H, Shamdasani V, Barth RA. Liver Fat Quantification by Ultrasound in Children: A Prospective Study. AJR Am J Roentgenol. 2021;217(4):996-1006.
